# Strategies to reengage patients lost to follow up in HIV care in high income countries, a scoping review

**DOI:** 10.1186/s12889-021-11613-y

**Published:** 2021-08-28

**Authors:** Jorge Palacio-Vieira, Juliana Maria Reyes-Urueña, Arkaitz Imaz, Andreu Bruguera, Luis Force, Amat Orti Llaveria, Josep M. Llibre, Ingrid Vilaró, Francesc Homar Borràs, Vicenç Falcó, Melchor Riera, Pere Domingo, Elisa de Lazzari, Josep M. Miró, Jordi Casabona, PICIS study group, Jordi Casabona, Jose M. Miró, Juliana Reyes, Andreu Bruguera, Sergio Moreno, Yesika Diaz, Jordi Aceiton, Esteve Muntada, J. Casabona, J. M. Miró, Sergio Moreno, Yesika Diaz, Jordi Aceiton, J. Reyes, E. Muntada, A. Bruguera, D. Podzamczer, A. Imaz, P. Domingo, J. M. Llibre, G. Navarro, C. Cortés, J. Mallolas, C. Manzardo, J. Tiraboschi, A. Curran, J. Burgos, M. Gracia Mateo, MM Gutierrez, J. Murillas, F. Segura, F. Homar, M. García-Gasalla, E. Gonzalez, F. Vidal, J. Peraire, L. Force, E. Leon, A. Masabeu, I. Vilaró, A. Orti, D. Dalmau, A. Jaen, A. Almuedo, E. De Lazzari, D. Giralt, B. Raventós, F. Gargoulas, T. Vanrell, J. C. Rubia, J. Vilà, M. Ferrés, B. Morell, M. Tamayo, J. Ambrosioni, M. Laguno, M. Martínez, J. L. Blanco, F. Garcia- Alcaide, E. Martínez, A. Jou, B. Clotet, M. Saumoy, A. Silva, P. Prieto, J. Navarro, E. Ribera, M. Gurgui, MA Ribas, A. A. Campins, F. J. Fanjul, M. Leyes, M. Peñaranda, L. Martin, H. Vilchez, S. Calzado, M. Cervantes, M. J. Amengual, M. Navarro, T. Payeras, C. Cifuentes, N. Abdulghani, T. Comella, M. Vargas, C. Viladés, P. Barrufet, Ivan Chivite, E. Chamarro, C. Escrig, M. Cairó, X. Martinez-Lacasa, R. Font, Sebastián Meyer, Juanse Hernandez

**Affiliations:** 1 Centre for Epidemiological Studies on Sexually Transmitted Infections and HIV/AIDS of Catalonia (CEEISCAT), Badalona, Spain; 2 CIBER Epidemiologia y Salud Pública (CIBERESP), Barcelona, Spain; 3 Hospital Clinic-Institut d’Investigacions Biomèdiques August Pi i Sunyer, University of Barcelona, Barcelona, Spain; 4 Institute for Health Science Research Germans Trias i Pujol (IGTP), Badalona, Spain; 5 HIV and STI Unit, Department of Infectious Diseases, Bellvitge University Hospital-IDIBELL, L’Hospitalet de Llobregat, Spain; 6 Internal Medicine, Hospital de Mataró-Consorci Sanitari del Maresme, Mataró, Spain; 7 Internal Medicine, Hospital Verge de la Cinta de Tortosa, Tortosa, Spain; 8 Infectious Diseases and “Fight AIDS” Foundation, University Hospital Germans Trias i Pujol, Badalona, Spain; 9 Hospital General de Vic, Vic, Spain; 10 Hospital Son Llàtzer, Palma de Mallorca, Spain; 11 Vall d’Hebron Hospital Universitari, Vall d’Hebron Institut de Recerca (VHIR), Universitat Autònoma de Barcelona, Barcelona, Spain; 12 Hospital Son Espases, Palma de Mallorca, Spain; 13 Hospital de Sant Pau, Barcelona, Spain; 14 HIV/AIDS Unit. Hospital Clinic, Barcelona, Spain

**Keywords:** Cohort studies, HIV, Lost to follow-up, Reengagement, Linkage

## Abstract

**Background:**

Despite remarkable achievements in antiretroviral therapy (ART), losses to follow-up (LTFU) might prevent the long-term success of HIV treatment and might delay the achievement of the 90–90-90 objectives. This scoping review is aimed at the description and analysis of the strategies used in high-income countries to reengage LTFU in HIV care, their implementation and impact.

**Methods:**

A scoping review was done following Arksey & O′Malley’s methodological framework and recommendations from Joanna Briggs Institute. Peer reviewed articles were searched for in Pubmed, Scopus and Web of Science; and grey literature was searched for in Google and other sources of information. Documents were charted according to the information presented on LTFU, the reengagement procedures used in HIV units in high-income countries, published during the last 15 years. In addition, bibliographies of chosen articles were reviewed for additional articles.

**Results:**

Twenty-eight documents were finally included, over 80% of them published in the United States later than 2015. Database searches, phone calls and/or mail contacts were the most common strategies used to locate and track LTFU, while motivational interviews and strengths-based techniques were used most often during reengagement visits. Outcomes like tracing activities efficacy, rates of reengagement and viral load reduction were reported as outcome measures.

**Conclusions:**

This review shows a recent and growing trend in developing and implementing patient reengagement strategies in HIV care. However, most of these strategies have been implemented in the United States and little information is available for other high-income countries. The procedures used to trace and contact LTFU are similar across reviewed studies, but their impact and sustainability are widely different depending on the country studied.

**Supplementary Information:**

The online version contains supplementary material available at 10.1186/s12889-021-11613-y.

## Background

Effective HIV antiretroviral therapy (ART) controls viral replication, enhances or maintains immune function and decreases morbidity and mortality, allowing people living with HIV (PLWH) to have a life expectancy comparable to general population and helps prevent new infections [1–3]. Several studies have demonstrated the effect of antiretroviral drugs in preventing HIV transmission by suppressing HIV RNA replication in people living with HIV (PLWH) to undetectable levels (treatment as prevention [TasP]), a strategy which has led to the ‘Undetectable equals Untransmittable’ (or ‘U=U’) campaign aimed at advocating for early access to HIV testing and treatment and motivate patients in the need to become virally suppressed through constant follow-up care [4]. Since it has been demonstrated that sexual transmission of HIV does not occur if undetectable blood plasma HIV RNA is maintained [5, 6], treatment for HIV also presents a great opportunity to reduce stigma and transform PLWH’s sexual relationships [7]. However, despite ART’s success, patient attrition and loss to follow-up (LTFU) remain legitimate threats to the long-term success of ART scale up.

The ambitious but achievable 90–90-90 UNAIDS target proposes that by 2020 90% of all PLWH know their HIV status, 90% of all people diagnosed HIV infection receive sustained antiretroviral therapy, and 90% of all people on ART maintain viral suppression (VS )[8]. In this sense, the main barriers in achieving this target in high income countries are patients lost during any of these three steps. Results from a multicentre study analysing 31 countries form Europe has shown that LTFU (defined as 12 months of HIV care disengagement) was 22%, and were mostly men, young, people who inject drugs and patients with high viral loads [9]. In another UK study, LTFU reached 28.1% of disengagement during nine consecutive months, and further analysis of these patients showed that 26% were found as transferred to clinics outside the UK [10]. In Spain, cohort studies reported 15% of LTFU (disengaged during a minimum of 12 months) and was linked to intravenous drug use, unemployment, more sporadic sex partners, being born in another country, and not having initiated ART [11]. In Catalonia and Balearic islands, the PISCIS cohort [12] has shown that 85% of HIV-diagnosed patients were retained (defined as 1 or more visits per year) [13] and a more recent study estimated that 89% of the PLWH were diagnosed, of these 78% were under treatment and 73% are virally suppressed [14]. Also in Catalonia, men who have sex with men (MSM) with a migration background experienced greater losses throughout the three steps of the 90–90-90 cascade: retention in care (74% vs. 55%), antiretroviral treatment (70% vs. 50%) and virally suppressed (65% vs. 46%), respectively [15].

In a step forward, USAID extended their target strategy to 95–95-95 by 2030 [16], which implies not only a need to increase retention in care, improve ART adherence and VS but also proactively reengage lost patients by hiring case managers to locate and give these patients the support they need to attend their clinical visits [17]. However, previous studies aimed at identifying the best measures to increase linkage, retention and reengagement to HIV care found no evidence-based interventions addressing reengagement [18] and described as major difficulties LTFU tracing, updating whereabouts data, lack of clinical records and time-gaps between HIV laboratory testing and initiating treatment [19]. The description and analysis of previous experiences will allow us to design, prepare and deploy a new evidence-based reengagement strategy directed at LTFU HIV positive patients in Catalonia and Balearic Islands, nested and piloted within a population-based cohort (PISCIS cohort), in order to help reduce the risk of untreated and disengaged HIV positive patients. The objective of this scoping review is to evaluate the current knowledge on LTFU patient reengagement, describe procedures used to re-link LTFU patients into treatment, and determine which factors play a role in the implementation of these strategies.

## Methods

Following scoping review methods described by Arksey H & O’Malley and the corresponding guidance developed by Peters et al. and Joanna Briggs Institute [20], we divided our review strategy into the following steps [21]:

- Stage 1: identify the research question

This review was guided by the question: “What is known about those interventions used to reengage patients in HIV treatment and reduce lost to follow-up?”

- Stage 2: identify relevant studies

Two types of documents were searched for: peer-review articles and grey literature. In the first case, searches were performed through PubMed, Scopus, Web of Science, Cochrane, PsycInfo EBSCO (see Additional file 1 for the search strategy). In the second case, grey literature was searched in Google. Initially, searches were performed using the following keywords in titles and abstracts, “reengagement/re-engagement”, “reengaged/re-engage”, “relink/re-link”, “HIV”, and “AIDS”; and then the medical subject headings (MeSH) were also used. All searchers where restricted to the last 15 years, (from January 2006 to May 2021) and were assessed by an independent librarian who checked the sources of data and the strategy used. In addition, bibliographies of chosen articles were further scanned for additional articles.

- Stage 3: study selection

Studies were selected if they fulfilled the following criteria: 1) defined, analysed, developed, or used HIV patient care reengagement strategies, 2) provided details on the type of strategies used, 3) showed details on the outcomes of its implementation, and 4) were carried out in high-income countries according to the classification of the World Bank [22]. Documents not published in English were excluded. Although, processes of search, selection and data charting were performed only by one person, one librarian specialist in health sciences research was consulted to assist and approve all these strategies.

- Stage 4: charting the data

Documents that met the inclusion criteria were registered in an Excel file specifying its title, author, abstract, intervention developed/used to reengage LTFU, type of study or design used, outcomes used to analyse the impact of the strategy, and key findings (see Additional file 2 for the final documents included in the review). Once chosen and entered into the study database, publications were revised again to confirm that they met inclusion criteria and complete missing information. In addition, the Preferred Reporting Items for Systematic Reviews - extension checklist for Scoping Reviews (PRISMA-ScR) was used (20] (see Additional file 3). Although the implementation protocol for this review was not registered, its objectives are encompassed within a broader project aimed at the study of LTFU in a cohort of PLWH in Spain.

## Results

**Fig. 1:**
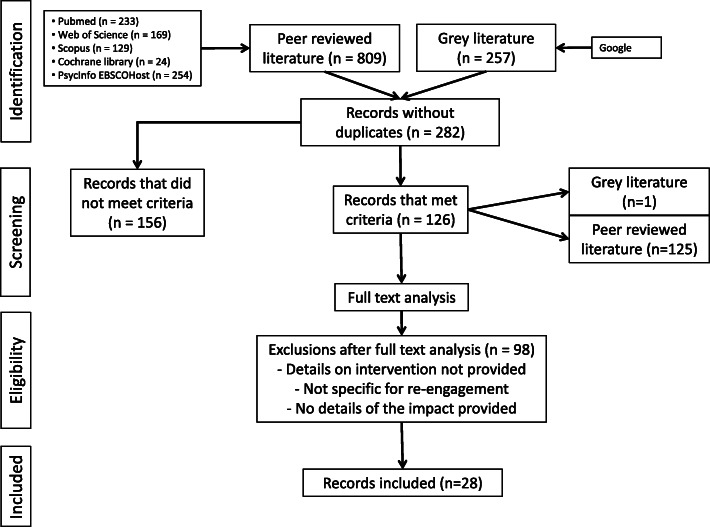
Flow diagram of data selection

A total of 282 documents were selected from both peer-review articles and grey literature sources and 126 were selected for full text assessment after reviewing their title and abstract. Twenty-eight documents fulfilled all the inclusion criteria. Figure 1 shows the review results and the number and characteristics of included and excluded articles. (Fig. 1, here).

### Studies characteristics

Our review yielded 27 peer-reviewed articles and one abstract presented in a virtual conference (Table 1). Most of the studies selected consisted in the use or test of an existing or slightly modified intervention (*n* = 16). The United States of America was the country of origin of 24 documents (86%) while Australia, Ireland, Trinidad Tobago and Canada presented 1 document each. Most documents were published in 2015 or after, while only 7% were published before. Exclusions were decided because published documents included data from middle- or low-income countries like Argentina, Puerto Rico, Kenya; other reported data on reengagement patients in care but did not show details of what kind of strategy they used or their efficacy or effectiveness.

**Table 1:** Characteristics of the documents included

Topic	n (%)	References
Type of publication		
Peer-reviewed article	27 (96)	[23–49]
Conference presentation	1 (4)	[50]
Design/type of study		
Retrospective patient tracing program	1 (4)	[30]
Registry-based analysis/prospective reengagement	1 (4)	[32]
Qualitative	1 (4)	[37]
Randomized controlled trial	2 (7)	[38, 41]
Comparative/case-control/assessment study	7 (25)	[25, 28, 33, 40, 42, 43, 49]
Using or testing an existing or slightly modified intervention	16 (56)	[23, 24, 26, 27, 29, 31, 34–36, 39, 44–48, 50]
Country		
Australia	1 (4)	[36]
Ireland	1 (4)	[42]
Trinidad and Tobago	1 (4)	[30]
Canada	1 (4)	[50]
United States of America	24 (86)	[23–29, 31–35, 37–41, 43–49]
Publication date		
Before 2015	2 (7)	[32, 44]
2015 or later	26 (93)	[23–31, 33–43, 45–50]

### Definitions of LTFU, professionals involved and contact methods

In order to consider patients as LTFU (some articles used the term “out-of-care patients” [23–28] to referred patients disengaged from care but are pending to be confirmed as lost) different time periods were reported in the reviewed literature, including at least six [23, 29], nine [28, 30–38], 12 [24–27, 39–45] and even 14 or 15 months since the last appointment to HIV care, CD4 cell count or viral load test [46, 47]. Different strategies to find LTFU patients were reported and, in most articles, they were used simultaneously. Depending on the complexity of LTFU patients, some were reengaged into HIV care was by means of a simple intervention, while others needed other more specialized services, like the referral to drug addiction units or the support of social, mental or prevention services [27–30, 38, 39, 45, 46]. Although diverse professionals were involved in the tracing and contact procedures, the most common categories were counsellors/patient navigators, while nurses and social workers were less common (Table 2).

**Table 2:** Methods and professionals involved in the LTFU patients’ reengagement

Intervention/Professional	Description (reference)	n (%)
Identification of LTFU and contacting method	- Distribution of flyers/brochures distributed to participants (29,38,44)	3 (10)
	- Coordination with other services (i.e. jails, pharmacies, hospitalization units) [25, 27, 28, 37, 38, 49]	6 (21)
	- Sending messages and contacting close relatives [24, 25, 30]	3 (11)
	- Home/field visit to the last known address [23–26, 28, 31–33, 48]	9 (32)
	- Internet searches and/or secondary database searches [25–28, 32, 33, 41, 45, 47–49]	11 (39)
	- Listing patients who meet LTFU criteria [23, 27, 30, 32, 34–37, 40, 41, 47, 50]	12 (43)
	- Post-mail letter/email [23–26, 28, 31, 34, 35, 40, 41, 43, 48, 49]	13 (46)
	- Use of electronic databases to confirm status [23–28, 30–36, 39–42, 45–50]	23 (82)
	- Calling patients by phone [23–38, 40–43, 46, 48–50]	23 (82)
Professionals involved (as reported in the records found)	- Nurses [50]	1 (4)
	- Social workers [30, 46]	2 (7)
	- Outreach coordinator [24, 43]	2 (8)
	- Peer mentor/partner advocates [29, 38, 48]	3 (8)
	- Disease intervention/linkage specialist [25, 28, 31, 41, 51]*	5 (21)
	- Not specified [35, 36, 40, 42, 44, 49]	6 (21)
	- Counsellors/patients navigators/field workers [23, 26, 27, 32–34, 37, 43, 47]	9 (24)

* In some cases, reengagement activities were shared among DIS and other professionals like Social Workers

Tracing and confirming the patient’s status was the initial way to determine if the patient is LTFU and according to the literature reviewed this was mainly achieved through the medical records [23–28, 30–36, 39–42, 45–50]. Patients were most classified as: a) patients in care (transferred to other HIV clinics, with up-coming appointments, incarcerated, moved to other cities or deceased) [23–26, 28, 30, 32, 34, 36, 37, 44]; or b) lost to follow-up or out of any HIV care or clinical follow-up. Three main types of interventions were then described: a) those that consisted solely on phone calls, letters/mails, text messages or home visits [23–25, 30, 43], b) others that started by establishing initial contact and were later complemented by a face-to-face intervention in the HIV clinic guided by a trained professional [26, 28, 29, 32–35, 41, 43] and c) a few opportunistic interventions addressed to out-of-care patients who were found in other medical units or contacted their health providers for different reasons not pertaining to HIV care [38, 40].

Phone calls were used to contact the patient in almost all reviewed articles [23–38, 40–43, 46, 48–50], with some studies specifying the number of calls made (for instance, a maximum of three calls) and the use of predesigned, tested scripts [25, 30, 33, 36]. When phone calls were not successful, other contact methods were reported, including mailing notifications (email or postal) to the last known address [23–26, 28, 31, 34, 35, 40, 41, 43, 48, 49], home visits [23–26, 28, 31–33, 48], searching for the patient in other public or external databases [25–28, 32, 33, 41, 45, 47–49] and contacting a close relative or their emergency contact [24, 25, 30].

In all interventions, professionals offered different procedures in order to reengage LTFU patients, such as identifying possible barriers/reasons for missed appointments and offering alternative solutions to facilitate reengagement and overcome those barriers [25, 26, 28, 30, 32, 33, 36, 39, 41, 42], rescheduling appointments [25, 26, 31–34, 42, 45] and giving further information on medication and alternatives to access treatment [25, 27, 29, 30, 38, 45, 46]. Other important strategy found were building report with the patient and enhancing patient empowerment [26, 29, 40, 41] by promoting the patient-centred care and setting goals for both patients and clinicians [27, 29, 31, 38] (Table 3).

**Table 3:** Interventions’ characteristics addressed to reengage patients to HIV care

Characteristic	Description (reference)
Details of the intervention	- Addressing patients with mental health, social needs or prevention services [27–30, 38, 39, 45, 46]
	- Comprehensive picture of the patient’s complete health/needs [26, 33, 36, 41, 48]
	- Promoting self-determination and self-care [27, 29]
	- Providing information on medication, access to care and treatment rights [25, 27, 29, 30, 38, 45, 46]
	- Building the staff-patient relationship and enhancing strengths [26, 29]
	- Knowledge and skills building [29, 38]
	- Schedule, change and complete a medical appointment [25, 26, 31–34, 42, 45]
	- Patient-centred care, setting objectives for patients and clinicians [27, 29, 31, 38]
	- Offering alternatives to complete appointments [23–26, 28, 31, 32, 36]
	- Identify and offering and solutions to the barriers to care [25, 26, 28, 30, 32, 33, 36, 39, 41, 42]
Theoretical background	- Social cognitive and wellness motivation theory [29]
	- Strengths-based case management/counselling [26–28, 33, 37, 47]
	- Motivational interview and behavioural skills [30, 38, 46–48, 50]
Length and time needed	- Three – ten phone calls [25, 30, 33, 49]
	- Two sessions of 20 to 44 min. Each followed by 5 phone calls during 10 weeks [38]
	- Six sessions (1 per week, during 6 weeks) + 1 booster session 6 weeks later [29]
	- One face-to-face interview of 45 min approximately [44, 46]
	- Two to ten sessions depending on the patients’ needs [26, 27]
	- Three field visits [33]

During reengagement visits, interventions were focused on encouraging active self-management and self-determination by means of the use of a conversational tone and often assisted by standard printed brochures. Patient-centred and tailored behavioural models like social cognitive theory and wellness motivation theory [30, 38, 46–48, 50], strengths-based case management/counselling and motivational interview and behavioural skills [26–28, 33, 37, 47] were some of the tools used during reengagement visits. According to the literature reviewed, the number of reengagement activities and amount of time invested on them is diverse (Table 3). While the majority of the articles used phone calls to trace LTFU, most did not specify details on the number of calls. A few cases consisted in three phone calls [25, 30, 33, 49], followed by sending one contact email, an attempt to reach patients through other databases, and finally reaching out to their designated emergency contact [25]. The number of reengagement visits with patients and their duration were also diverse, with reports ranging between two sessions of 20 to 60 min [38, 42], up to five [26, 27] or six sessions (1 per week, during 6 weeks) with a booster session 6 weeks later [29].

### Efficacy of the strategies analysed

Several outcomes were used to measure the reengagement activities (Table 4). First outcome, although it is not specifically related to the reengagement process, was the proportion of patients found to be in care in other centres. Between 2 and 46% of the patients who were initially considered as LTFU but were found to be transferred to other HIV care [24, 30, 34, 36, 40, 41, 48, 49]. Second, the proportion of patients that were successfully in contacted varied widely, ranging between 19 and 98% [25, 28, 30, 32, 33, 42, 46–50]. Thirdly, the percentage of successful interventions, considered those that made LTFU patients return to regular care and restart ART. Relinkage to care or restarting treatment ranged between 7 and 86% [23–26, 28–36, 39–50]. Finally, some publications reported long-term outcomes, such as Viral Suppression (VS) at 6 months (34–90%) [29, 34, 41, 46] and 12 months (30–67%) [23, 29, 33, 34, 39, 40, 47] since the intervention. Other middle-long-term outcomes consisted in analysing retention after 1 to 6 months after the intervention [26, 30, 39]. Qualitative studies showed that PLWH described the work of navigators as supportive and helpful to find alternatives of care and these professionals described their work with clients as important to identify individual needs and behaviours that could improve PLWH care [37].

**Table 4:** Assessment methods and results of the activities reported to reengage patients to HIV care

Reengagement activities and outcomes	Measurement (median)	Reference
Patients’ tracing activities and status confirmation		
In HIV care/misclassified as LTFU	11–61% (33%)	[23, 25, 26, 28, 31, 32, 34, 36, 46–48, 50]
Transferred to another clinic/care	2–46% (12%)	[24, 30, 34, 36, 40, 41, 48, 49]
Deceased	0.6–13% (6%)	[23, 24, 26, 28, 30, 31, 34, 36, 40, 41, 48–50]
Migrated/changed city	5–51% (12%)	[25, 26, 30, 31, 40, 41, 48, 49]
Incarcerated/other outcomes	1–9% (4,5%)	[24, 30, 32, 40]
Not located/unreached/not contacted	3–81% (29%)	[24–26, 28, 31, 32, 34, 36, 40–42, 47–49]
Contacted	19–98% (47%)	[25, 28, 30, 32, 33, 42, 46–50]
Reengagement outcomes		
Returned/linked to care/restarted treatment	7–86% (56%)	[23–26, 28–36, 39–50]
Refused linkage to care	3–58% (12%)	[26, 30–32, 36, 41, 47–50]
Follow-up outcomes		
Viral suppression at 6 months	34–90% (66%)	[29, 34, 41, 46]
Viral suppression at 12 months or more	30–67% (58%)	[23, 29, 33, 34, 39, 40, 47]
Retained within 1–6 months	28–82% (55%)	[26, 30, 39]
Retained within 1 a year	50–90% (56%)	[23, 24, 39, 43, 47]
2 visits/laboratory tests	22–72% (50%)	[38, 49]

### Determinants of the intervention and LTFU’ characteristics

Different variables are seen to be associated with successful interventions. In the United States, patients with Asiatic background were more likely to be reengaged than blacks [30, 33, 43]. Age and gender are also associated with successful reengagement and retention, with some studies showing that younger patients are less reengaged than those over 40 years of age [23, 25, 33] and males reengaged less than women [24, 44]. Steady income, the ability to meet basic needs (food and housing), having a reliable cell-phone, and accurate contact information (as these programs rely on making direct contacts) are related with better outcomes in retention after the implementation of reengagement strategies [25, 42]. On the contrary, barriers such as the lack of transportation, unstable housing, the lack of financial or material resources, mental health, and substance abuse issues [28, 44], scheduling issues, and feeling stigmatized were associated with no reengagement [30]. Compared to MSM, other key populations are less likely to achieve VS after reengagement, especially people who inject drugs, heterosexuals, or those not in any of these key populations [23]. In comparison to those aged 18–29, PLWH who were 40–49 years showed an increased likelihood of VS within two different periods of time since the intervention, 180 days [34] and 1 year [33]. LTFU patients with higher viral loads and assigned to an intervention strategy were less likely to reengage in care within 180 days and to achieve VS within 1 year of the intervention, compared to patients who were not assigned to the intervention [28]. In other studies re-engagement was associated with having reached viral suppression by the time of the inclusion in the implementation strategy [39]. It is important to mention that one of the articles reviewed, consisted in a cluster randomized evaluation of a intervention designed to Increase reengagement did not showed differences between the patient subgroups assessed [41].

## Discussion

Retention in care and ART adherence constitute key strategies in HIV management and are essential for PLWH in order to stay healthy, live longer and prevent HIV transmission to others. Although few publications fulfilled the inclusion criteria for this scoping review, the selected studies provide useful and well organized information on how to identify LTFU patients, how to implement successful strategies to motivate and reengage patients into HIV care, and how to determine the impact of such strategies. During the identification of LTFU patients, electronic databases and other data sources are useful to obtain information on probable patients, and phone calls were the most used strategy in contacting these patients, combined with mailed letters, emails, home visits. The reengagement process itself is performed mainly by finding and reducing the barriers that make patients disengage and enhancing their motivation to return to HIV care, while preventing new losses.

Most of the selected studies were published in the United States and only one study was European (Ireland), evidencing a lack of knowledge in this field in different contexts. Analysing reengagement strategies under different HIV-care, social, and economic factors is still pending, and this would imply adapting the different strategies to local circumstances, testing which of them would work well, and measuring their impact on the reengagement of LTFU patients. Additionally, the fact that most of the studies were published after the year 2015 shows a growing need in reaching this group of PLWH, that despite their particular circumstances, could easily return to treatment and improve their health, with a relatively low effort and few resources invested. The dynamism of the HIV epidemic and the increased interest in LTFU patient reengagement are aligned with the treatment and care objectives (90–90-90 and 95–95-95), which not only focus in maintaining low viral loads and well controlled patients, but also in reducing diagnostic and treatment gaps.

Defining who is a LTFU patient is crucial, and the most used classification found is having no contact with HIV care professionals for over 12 months. However, why this is or other time-periods were chosen is only described in one of the reviewed articles, referring to specific clinical guidelines [36]. Selecting 12 months or longer periods was described as more challenging due to the difficulties in acquiring patients’ contact information the longer they’ve been out-of-care [37]. Shorter periods, such as 6 months, were selected due to clinical reasons [34], because patients that were out-of-care during this period of time showed reduced chances of reengagement, which according to the authors may be explained by treatment regimen fatigue, and decreased motivation and adhere to medication as prescribed [23]. In contrast, longer periods of time to be considered as out-of-care were also reported, such as 14 months, because authors tried to minimize the number of patients falsely identified as being out of care due to delays in data reporting, maximizing the specificity of the classification strategy [46]. Standard definitions of retention in care in the United States are two clinical visits separated by ≥90 days during a 12-months measurement period or at least 1 visit in each 6-months period during a 24-months measurement period, with ≥60-d between visits in adjacent 6-mo periods [52]. This might have implications for the organization, provision of HIV care and the need to follow patients frequently and avoiding losing them for even 6 months.

The most common way to find LTFU patients was to look for them in the clinical records, with some authors reporting the use of multiple data bases, as well as using external or secondary sources of information. As the majority of the studies were published with data from United States, where patients can be registered with different healthcare or buy-in providers [23], combining different sources of information was common in order to locate patients. However, in certain European countries, where healthcare systems have less access barriers by having integrated and connected health records, tracking LTFU patients and reengaging them into HIV care could take less time and resources.

Tracking activities consisted mainly in making phone calls and sending letters/emails, with some publications also referring home visits to the patient’s last known address, but few details on how confidentiality was ensured during home visits was described. Phone calls were reported to be successful in reengaging patients, even when used alone, and authors mentioned the potential benefit of using this method in other countries/settings given its relatively low-cost and little time needed [24]. The type, quantity, and time spent in reengagement procedures was also quite diverse among the reviewed literature, and there was no common trend since each study presented specific circumstances. However, one publication mentioned that the length of the intervention period was probably the reason of unsuccessful results [38] and another study modified an intervention to include more sessions and offered linkage to other healthcare services [26].

There was a considerable number of PLWH that, despite multiple efforts, could not be reached and reengaged. In the Irish study, where 81% of the contacts were reported to be unsuccessful, authors, along with others, explained that out-of-service phone numbers might explain for this high proportion and highlighted the need to maintain updated demographic and contact information within each visit [30]. To do this, a strong and multidisciplinary work-team needs to be built within each centre, or even between multiple centres, in order to manage and update contact information and keep patients’ registries as accurate as possible, avoiding wrong classifications of LTFU and optimizing the time needed to attend patients [25–27, 30, 33]. Updated clinical records is crucial, and some studies pointed out that at least a third of the presumed LTFU patients might actually be in care, reinforcing the importance of having a strong system that keeps these patients as well tracked as possible [32, 37]. Moreover, integrating different records, for instance clinic-based and administrative records could avoid losses and ease the implementation of reengagement activities [31]. In fact, these strategies has been reported as an opportunity for improving clinical registries, allowing at the same time the proper follow-up of patients [25]. In addition, the proper use and sharing clinical records might help avoid extra work in finding presumably lost patients, when they might have simply transferred to other clinics, which according to the reviewed literature, can be as much as 50% of all LTFU. The implementation of field/outreach programs aimed at identifying, tracing, following-up on, and reengaging LTFU patients can complement other clinic staff’s efforts to locate patients who are hard to reach or those whose present access and adherence to care barriers such as lack of transportation, unstable housing, poverty, mental health, substance use disorders, among others [24, 28].

There is evidence on how some determinants interfere in the likelihood of being successfully reengaged in HIV care. The provision of evidence-based training to health professionals and the improvement of the communication skills would enhance the chance of reengagement, reducing also the risk of stigma, time limitations and other difficulties [30, 41]. In addition, reengagement strategies should consider the fact that low socioeconomic status plays an important role, making reengage less likely [28] which in turn increases the need for social workers to collaborate with HIV care and find better ways to coordinate the units staff [24]. Clinical variables have also been associated with reengagement. High levels of CD4 cell counts and lower viral loads have been reported as determinants of higher likelihood of reengagement [24]. When patients have returned to care and are followed over time (90 or 180 days), viral loads remain lower compared to those not reengaged and retained [34]. Similarly, being diagnosed with either concurrent or nonconcurrent AIDS increases the likelihood of being retained in care after reengagement [23].

Successful reengagement depends largely on the ability of the professional to involve and motivate patients to attend future appointments and working with them to solve the specific reasons why they may disengage. Additionally, the number and length of these strategies, and the efforts made to achieve success, might depend on the professionals’ ability to tailor and modify these strategies according to each particular circumstance [26]. Strength and motivational-based strategies are common among the revised studies, and basically consist in finding out and collaborating with the patient in order to find the best options in dealing with each particular engagement barrier. The role of the healthcare professionals is very important, especially in motivating LTFU patients, so professionals should be trained on increasing their ability to meet the patients’ needs, listening and working together to find solutions and overcoming the barriers that kept them out of care [41]. Although the professionals’ work should be as holistic as possible, some LTFU patients could have needs which are difficult to meet. However, there is a basic group of LTFU needs that can ensure reengagement, which include: prioritization of urgent needs, dividing needs into short and long term, relying on partner organizations to provide support services, and having priority status to be included in programs like addiction units or social work assistance [37].

A limitation of our review is the fact that only articles published in English were searched, missing possibly relevant information in other languages. In addition, although searches were performed to include different types of documents, the vast majority of the results were related to peer-reviewed articles and almost no reports or conference proceedings were found.

## Conclusions

The new targets related with the control of the HIV epidemic, consisting in reducing the gap of treatment adherence and retention in care, possess new challenges for healthcare providers. If the new target 95–95-95 treatment challenges for HIV care are to be met, health care systems need to adapt themselves and proactively address LTFU patient reengagement and retention efforts. Although the development of reengagement strategies is relatively new and there are certain tasks in common in the literature, there is still lack of information about how these strategies work in different contexts, for instance in Europe. Most of the evidences on the procedures, methods, and impact of reengagement strategies come from United States, where barriers and determinants could be different to other countries. This review shows that certain procedures, like the definition of who is LUTF, the patient tracing in different databases and the use of different contact strategies might have a significant impact on the reengagement of LTFU, but they largely depend on the quality of the data available of these patients. The results and lessons learned from this review have several implications on the provision and management of HIV care systems, these results can serve as a guide to formulate future reengagement strategies so as to reach HIV care goals adapted to different contexts.

## Supplementary Information

**Additional file 1.** Sources and search strategy used.

**Additional file 2.** List and details of included papers.

**Additional file 3.** Preferred Reporting Items for Systematic reviews and Meta-Analyses extension for Scoping Reviews (PRISMA-ScR) Checklist.

## Data Availability

The datasets supporting the conclusions of this article are included within the article.

## Abbreviations

AIDS: Acquired immunodeficiency syndrome
ART: Antiretroviral therapy
CD4: Cluster of differentiation 4
HIV: Human immunodeficiency virus
LTFU: Lost to follow-up
MSM: Men who have Sex with Men
PLWH: People living with HIV
USAID: United states agency for international development
VS: Viral suppression
