# Supplementary material for: Strategies to reengage patients lost to follow up in HIV care in high income countries, a scoping review
Source: BMC Public Health. 2021 Aug 28;21:1596. doi: 10.1186/s12889-021-11613-y (PMC8403456; doi:10.1186/s12889-021-11613-y)
Supplement: Supplementary file 1 — Additional file 1. Sources and search strategy used. [file 12889_2021_11613_MOESM1_ESM.docx]

**Additional file 1:** Sources and search strategy used.

**PubMed**

| **Search** | **Query** | | | **Results** |
| --- | --- | --- | --- | --- |
| #6 | (("reengag*"[Title/Abstract] OR "relink*"[Title/Abstract] OR "re engag*"[Title/Abstract] OR "re link*"[Title/Abstract])) AND (((AIDS[MeSH Terms]) OR (HIV[MeSH Terms])) OR ((AIDS[Title/Abstract]) OR (HIV[Title/Abstract]))) Filters: from 2006 - 2021 Sort by: Publication Date | | | 233 |
| #5 | #4 AND #3 | | | 239 |
| #4 | ("reengag*"[Title/Abstract] OR "relink*"[Title/Abstract] OR "re engag*"[Title/Abstract] OR "re link*"[Title/Abstract]) | | | 1,129 |
| #3 | #1 OR #2 | | | 428,209 |
| #2 | (AIDS[MeSH Terms]) OR (HIV[MeSH Terms]) | | | 167,820 |
| #1 | (AIDS[Title/Abstract]) OR (HIV[Title/Abstract]) | | | 405,594 |
|  | **Results** | | |  |
|  | Screened | Assessed | Included |  |
|  | 181 | 70 | 27 |  |

**Cochrane library**

| **Search** | **Query** | | | **Results** |
| --- | --- | --- | --- | --- |
| #1 | (HIV):ti,ab,kw AND (Reengagement):ti,ab,kw | | | 24 |
|  | **Results** | | |  |
|  | Screened | Assessed | Included |  |
|  | 3 | 1* | 0 |  |

* Duplicate with Pubmed

**Web of Science**

| **Search** | **Query** | | | **Results** |
| --- | --- | --- | --- | --- |
| #1 | TOPIC: (HIV, re-engagem*) OR TOPIC: (HIV, reengage*)  Timespan: 2006-2021. Databases: WOS, BIOSIS, CCC, DIIDW, KJD, RSCI, SCIELO. Search language=Auto | | | 169 |
|  | **Results** | | |  |
|  | Screened | Assessed | Included |  |
|  | 34 | 22* | 0 |  |

* Duplicate with Pubmed

**PsycInfo (EBSCOHost)**

| **Search** | **Query** | | | **Results** |
| --- | --- | --- | --- | --- |
| #1 | hiv AND reengagement OR re-engagement (2006-2021) | | | 254 |
|  | **Results** | | |  |
|  | Screened | Assessed | Included |  |
|  | 20 | 12 (duplicated with Pubmed) | 0 |  |

* Duplicate with Pubmed

**Scopus**

| **Search** | **Query** | | | **Results** |
| --- | --- | --- | --- | --- |
| #1 | ( TITLE-ABS-KEY ( hiv ) AND TITLE-ABS-KEY ( reengagem* ) OR TITLE-ABS-KEY ( re-engagem* ) ) | | | 129 |
|  | **Results** | | |  |
|  | Screened | Assessed | Included |  |
|  | 15 | 10* | 0 |  |

* Duplicate with Pubmed

**Google**

| **Search** | **Query*** | | | **Results** |
| --- | --- | --- | --- | --- |
| #1 | HIV, re-engagement, -Africa | | | 151 |
|  | Screened | Assessed | Included |  |
|  | 20 | 7** | 1 |  |
| #2 | HIV, reengagement, -Africa | | | 106 |
|  | Screened | Assessed | Included |  |
|  | 9 | 4** | 0 |  |
|  | **Results** | | | |
|  | Screened | Assessed | Included |  |
|  | 29 | 11 | 1 |  |

*Ordered by relevance and only first 400 documents screened.

** Duplicate with Pubmed
